# Effectiveness of virtual reality based intervention on core symptoms and cognitive functions in attention−deficit hyperactivity disorder: a systematic review and meta−analysis

**DOI:** 10.3389/fpsyg.2026.1835922

**Published:** 2026-08-07

**Authors:** Huaibo Yang, Yuncong Zhou, Chengyu Zhou, Diandong Lang, Li Chen

**Affiliations:** 1 Department of Physical Education, Nanjing University of Finance and Economics Hongshan College, Nanjing, China; 2 Department of Physical Education and Research, China University of Mining and Technology, Beijing, China; 3 Strength and Conditioning Training College, Beijing Sports University, Beijing, China; 4 School of Physical Education and Health, Linyi University, Linyi, China; 5 Department of Physical Education Teaching, Shanghai Sanda University, Shanghai, China

**Keywords:** attention deficit hyperactivity disorder, cognitive function, core symptoms, meta−analysis, systematic review, virtual reality intervention

## Abstract

**Objective:**

This systematic review and meta−analysis aimed to comprehensively evaluate the impact of virtual reality (VR)−based interventions on core symptoms and cognitive function, and to examine their effectiveness for Attention−Deficit/Hyperactivity Disorder (ADHD).

**Methods:**

A comprehensive search was conducted across multiple databases, including Embase, Web of Science (WOS), PubMed, The Cochrane Library, ProQuest, EBSCOhost, from their inception up to May 2026. The search aimed to identify randomized controlled trials (RCTs) investigating the impact of VR on core symptoms and cognitive function in patients with ADHD. The Physiotherapy Evidence Database (PEDro) scale was employed to assess the quality of the literature, while the revised Cochrane risk−of−bias tool (ROB−2) was used to evaluate the overall risk of bias. The Grading of Recommendations, Assessment, Development and Evaluation (GRADE) profiler method was utilized to further assess the quality of evidence. Meta−analysis, sensitivity analysis, and publication bias testing were performed using Review Manager 5.4.1 and Stata 18.0 software. Effect sizes were calculated using the standardized mean difference (SMD) and 95% confidence intervals (CI).

**Results:**

23 RCTs with 1,102 participants were included. Meta−analysis showed that VR interventions significantly reduced core ADHD symptoms [inattention: SMD = −0.80, 95% CI (−1.13, −0.48), *p* < 0.05, *I^2^* = 79%; hyperactivity/impulsivity: SMD = −0.57, 95% CI (−0.87, −0.27), *p* < 0.05, *I*^2^ = 76%; total symptoms: SMD = −0.47, 95% CI (−0.78, −0.16), *p* < 0.05, *I*^2^ = 54%],and significantly improved cognitive functions [inhibition: SMD = −0.36, 95% CI (−0.56, −0.15), *p* < 0.05, *I^2^* = 37%; working memory: SMD = −0.23, 95% CI (−0.40, −0.07), *p* < 0.05, *I*^2^ = 0%; cognitive flexibility: SMD = −0.23, 95% CI (−0.43, −0.04), *p* < 0.05, *I^2^* = 0%]. Subgroup analysis revealed that for children under 12 years of age, mid−term (5–8 weeks), moderate−duration (20–30 min), high−frequency (≥3 sessions/week) interventions using mixed cognitive−physical or cognitive−based VR significantly improve core symptoms in patients with ADHD, with immersive VR producing more consistent broad symptom relief; cognitive functions require long−term (≥9 weeks) interventions with long duration (>30 min) per session. Lower frequency (1–2 sessions/week) appears more beneficial than higher frequency, and non−immersive VR may be more effective than immersive for inhibition and working memory. Cognitive improvements are most evident in children under 12 years.

**Conclusion:**

VR interventions significantly reduce core ADHD symptoms and improve cognitive functions, with differential dose–response patterns. Optimal protocols differ between symptoms and cognitive outcomes. VR represents a promising complementary approach for ADHD, pending confirmation through high−quality, long−term RCTs.

**Systematic review registration:**

https://www.crd.york.ac.uk/prospero/, identifier PROSPERO (CRD420261340074).

## 1 Introduction

Attention−Deficit/Hyperactivity Disorder (ADHD) affects 5–7% of children globally, characterized by inattention, hyperactivity, and impulsivity that impair daily functioning (Salari et al., 2023). Beyond core symptoms, children with ADHD exhibit significant cognitive deficits, including impairments in processing speed (*d* = −0.55), working memory (*d* = −0.53), response inhibition (*d* = −0.64), and attentional vigilance (*d* = −0.71) compared to typically developing peers (Pievsky and McGrath, 2018; Huang−Pollock et al., 2012; Willcutt et al., 2005). These deficits affect 40–60% of affected children and independently contribute to adverse academic and occupational outcomes, underscoring the importance of targeting both symptom domains and cognitive processes in intervention (Biederman et al., 2004; Faraone et al., 2021).

Current treatment approaches for ADHD include pharmacological interventions, primarily stimulant medications, which demonstrate robust efficacy in symptom reduction (Ostinelli et al., 2025). However, concerns regarding side effects, variable treatment response, and parental preferences for non−pharmacological options necessitate the development of effective alternative interventions (Arnold et al., 2015). Non−pharmacological approaches, including behavioral therapy, cognitive training, and neurofeedback, have shown promise but face challenges related to treatment adherence, skill generalization, and variable effect sizes (Dovis et al., 2015; Machado et al., 2025).

Virtual reality (VR) technology has emerged as a promising tool in mental health interventions across various conditions (Cao et al., 2026). VR enables the creation of standardized, ecologically valid environments that simulate real−world demands while maintaining experimental control, offering unique advantages including enhanced patient engagement, real−time feedback, and graded challenge delivery (Machado et al., 2025; Riva and Serino, 2020; Parsons and Rizzo, 2008). VR−based interventions have demonstrated efficacy in treating anxiety disorders, phobias, post−traumatic stress disorder, and in cognitive rehabilitation among various populations (Machado et al., 2025). Current clinical practice primarily relies on a combination of pharmacological and non−pharmacological interventions for ADHD. However, pharmacotherapy is associated with side effects and adherence issues, while traditional non−pharmacological interventions face challenges such as low engagement and insufficient personalization, highlighting an urgent need for innovative treatment approaches. Virtual reality (VR) technology, characterized by high immersion, strong ecological validity, and multi−sensory dynamic interaction, can simulate real−world environments and enable individualized training, thereby representing a promising research direction for non−pharmacological interventions in ADHD (Cao et al., 2026).

However, the application of VR specifically for improving core ADHD symptoms and cognitive functions in children remains limited, and existing studies have yielded inconsistent findings. Some studies report improvements in attention performance within simulated classroom environments, while others find no significant effects on objective neuropsychological measures (Pérez−Rodríguez et al., 2025; Zheng et al., 2025). In addition, no meta−analysis to date has simultaneously examined effects on both core ADHD symptoms and cognitive functions.

Therefore, this study aimed to conduct the first systematic review and meta−analysis focused on identifying effectiveness of VR−based interventions on decreasing core symptoms and improving cognitive functions in children with ADHD. Given randomized controlled trials (RCTs) are recognized as the gold standard design for exploring the effectiveness whilst non−RCTs produce more research bias (Shrier et al., 2007), this study restricting inclusion to RCTs to ensure high internal validity.

## 2 Methods

This systematic review was prospectively registered with the National Institute for Health Research website PROSPERO. Details of the protocol can be accessed at: https://www.crd.york. ac.uk/Prospero/Record ID, CRD420261340074.

### 2.1 Search strategy

This study was reported in accordance with the Preferred Reporting Items for Systematic Review and Meta−analysis (PRISMA) guidelines (Moher et al., 2009). Six databases, including Embase, Web of Science (WOS), PubMed, The Cochrane Library, ProQuest, EBSCOhost, were searched from their inception to May 2026. The following categories of keywords were developed: population−related, intervention related, and outcome related. The detailed search strategy is provided in Supplementary Appendix S1.

### 2.2 Inclusion and exclusion criteria

Inclusion criteria included: 1) participants in this study comprised children, adolescents, and adults; 2) samples with attention deficit hyperactivity disorder (ADHD); 3) a randomized con−trolled trial (RCT); 4) virtual reality (VR) component; 5) core symptoms and/or cognitive function as outcome(s); and 6) published in English in peer−reviewed journals. On−going studies and review articles were excluded. After duplicates were removed, title and abstract screening and full−text screening were conducted according to the selection criteria. Reference lists of the included studies and relevant reviews were manually checked to identify additional studies. Two authors selected and reviewed articles in parallel. The corresponding author made final decisions once there were any disagreements.

### 2.3 Literature screening and data extraction

All retrieved records were imported into EndNote X9 for duplicate removal. Two independent reviewers (DL and LC) screened the studies according to the predefined inclusion and exclusion criteria, extracted relevant data, and cross−checked the results. Any disagreements were resolved through discussion with a third reviewer (YZ) to reach a consensus on study inclusion.

In addition, the following procedures were applied to specific cases: (1) when studies reported outcomes only in graphical form without providing raw data, and original data could not be obtained after contacting the authors, data were extracted using a digital plot extraction tool (Song et al., 2024); (2) when multiple publications originated from the same study and reported identical or overlapping outcomes, only the most recently published article was included (Chen et al., 2025); and (3) data were extracted using a standardized data extraction form.

The extracted information included: (1) basic study characteristics (country of study, first author, and year of publication); (2) study design and participant characteristics, including sample size per group, age, type of intervention, intervention duration, total number of sessions, weekly frequency, and session length; (3) outcome measures (working memory and anxiety/depression); (4) mean values and standard deviations of outcome measures before and after the intervention.

### 2.4 Assessment tools for the outcome indicators

The primary outcome was core symptoms (inattention, hyperactivity/impulsivity, total symptoms), and the secondary outcomes were cognitive function (inhibition, working memory, cognitive flexibility). These symptoms scores were assessed using standardized self−report questionnaires, clinician−rated scales, or behavioral tasks. The assessment instruments primarily included the Swanson, Nolan, and Pelham Questionnaire (SNAP−IV); Behavior Rating Inventory of Executive Function (BRIEF); Frankfurter AufmerksamkeitsInventar (FAIR:); Advanced Test of Attention (ATA); Sequence of Letters and Numbers (SLN); Flanker Task reaction times (FTRT); ADHD VragenLijst (ALA); The continuous performance task (CPT).

### 2.5 Risk of bias assessment

Risk of bias was assessed using the Revised Tool to Assess Risk of Bias in Randomized Trials (RoB 2.0) (Zhang et al., 2025). This tool evaluates five domains, including the randomization process, deviations from the intended interventions, missing outcome data, measurement of the outcome, and selection of the reported results (Sterne et al., 2019). Each domain was assigned with a “low,” “high,” or “some concerns” risk judgment. A judgment of a “high” risk of bias for any individual domain led to the result being at “high” risk overall, and a judgment of “some concerns” for any individual domain led to the result being at “some concerns,” or “high” risk, overall (Sterne et al., 2019). The assessment was conducted independently by two authors; the corresponding author made the final decisions when there were any disagreements.

### 2.6 Quality assessment of evidence

The GRADE profiler system (Guyatt et al., 2008) was used to assess the quality of evidence for the outcome indicators. This assessment considered five factors that may lead to downgrading: risk of bias, inconsistency, indirectness, imprecision, and publication bias. The quality of evidence was classified into four levels: high (no downgrading), moderate (downgraded by one level), low (downgraded by two levels), and very low (downgraded by three levels). Two researchers independently performed the quality assessment. In the event of disagreement, a third researcher was consulted to reach a consensus through discussion.

### 2.7 Statistical analyses

Review Manager (version 5.4.1) software was employed for effect size combination, subgroup analysis, sensitivity analysis. The standardized mean difference (SMD) and 95% confidence interval (CI) were utilized to combine effect sizes, accounting for variations in testing methods and measurement units. SMD values were categorized as fair (<0.2), small (0.2–<0.5), moderate (0.5–<0.8), and large (≥0.8). Heterogeneity was assessed using Higgins’ *I^2^* statistic, with *I^2^* ≤ 25% indicating no heterogeneity, 25% < *I^2^* ≤ 50% low heterogeneity, 50% < *I^2^* ≤ 75% moderate heterogeneity, and *I^2^* > 75% high heterogeneity. Studies were considered homogeneous and analyzed using a fixed−effect model if *I^2^* < 50% and *p* > 0.1. Further determination of heterogeneity sources was necessary if *I^2^* ≥ 50% and *p* < 0.1. Sensitivity analyses were conducted when *I^2^* exceeded 50% to assess the robustness of merged results (Song et al., 2024; Chen et al., 2025).

## 3 Results

### 3.1 Search results

**Figure 1:**
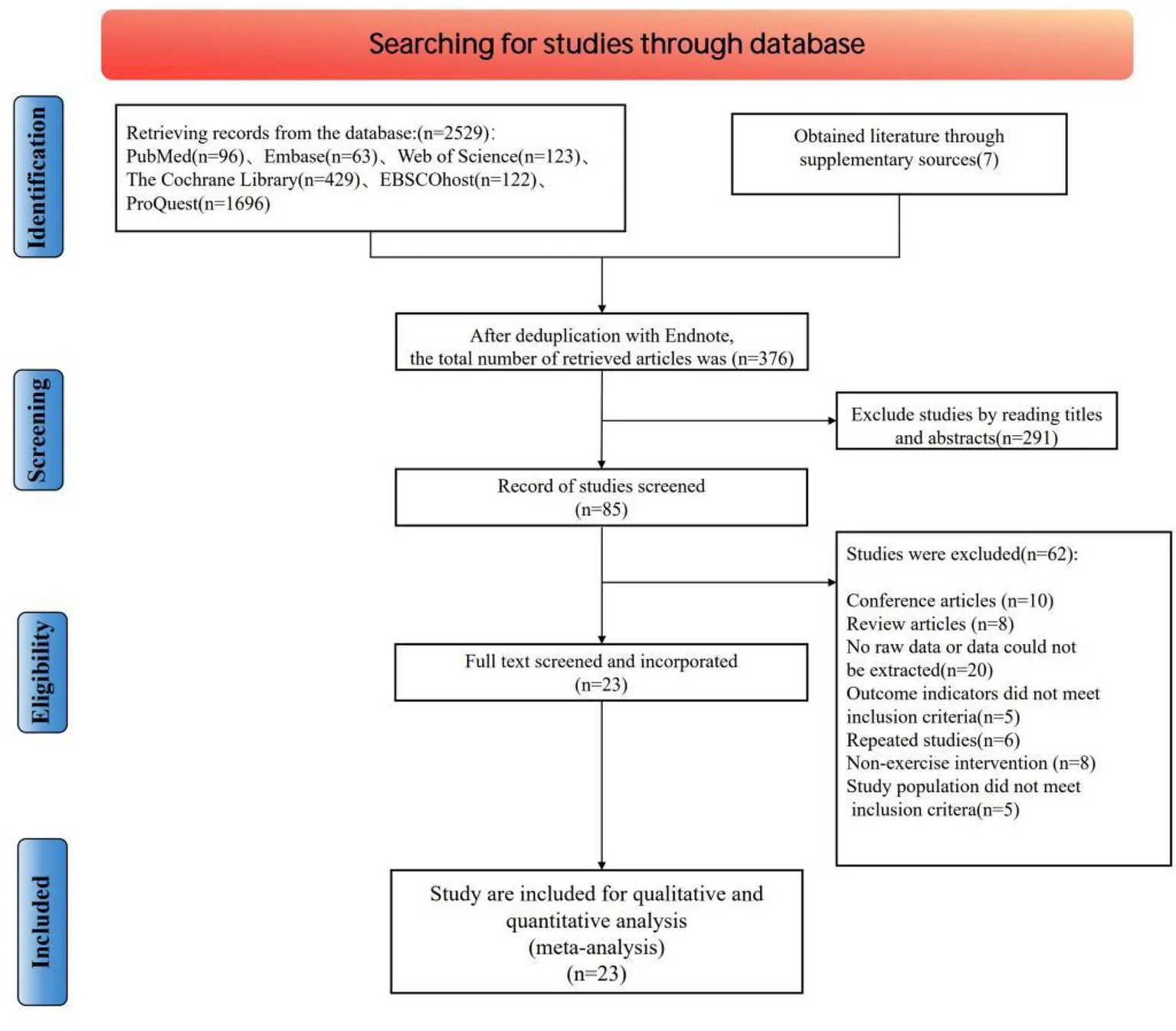
Selection of study and screening flowchart as required by the PRISMA statement.

A comprehensive literature search yielded 2,529 articles from various databases: PubMed (*n* = 96), Embase (*n* = 63), Web of Science (*n* = 123), The Cochrane Library (*n* = 429), EBSCOhost (*n* = 122), ProQuest (*n* = 1,696). An additional 7 articles were manually identified. A total of 376 records were identified after importing all retrieved studies into Endnote and removing duplicates. Following the initial screening of titles and abstracts, 85 articles were selected for further evaluation. Subsequent full−text review led to the exclusion of 62 articles for the following reasons: conference articles (*n* = 10), review articles (*n* = 8), absence of raw data or inability to extract data (*n* = 20), outcome measures not meeting the inclusion criteria (*n* = 5), duplicate studies (*n* = 6), non−exercise interventions (*n* = 8), and study populations not meeting the inclusion criteria (*n* = 5). Ultimately, 23 studies were included in the meta−analysis. Figure 1 provides a detailed illustration of the literature screening process.

### 3.2 Basic information and intervention characteristics of the included studies

The analysis included 23 RCTs (Dovis et al., 2015; Lee et al., 2001; Cho et al., 2002; Cho et al., 2004; Weerdmeester et al., 2016; Bikic et al., 2017; Hahn−Markowitz et al., 2017; Hudak et al., 2017; Benzing et al., 2018; Bikic et al., 2018; Bioulac et al., 2018; Benzing and Schmidt, 2019; Kim et al., 2020; Skalski et al., 2021; Chang et al., 2022; Cunha et al., 2023; Ji et al., 2023; Molazamani et al., 2023; Rodrigo−Yanguas et al., 2023; Kim et al., 2024; Martin−Moratinos et al., 2024; Wong et al., 2024; Zhao et al., 2024), encompassing a total of 1,102 participants. The subjects were predominantly children and adolescents diagnosed with ADHD, ranging from 6 to 22 years of age, with studies published between 2001 and 2025. All included studies implemented virtual reality (VR) or digital intervention for the experimental group. Regarding the level of immersion, 12 studies (52.2%) employed immersive VR, while 11 studies (47.8%) used non−immersive VR. In terms of intervention types, cognitive−based interventions were the most prevalent (17 studies, 73.9%), followed by physical activity−based interventions (5 studies, 21.7%), and mixed cognitive−physical interventions (1 study, 4.3%). The duration of individual sessions varied from 10 to 60 min, with the most common session length being 30 min (8 studies, 34.8%). Intervention frequency ranged from 1 to 6 sessions per week, with weekly frequencies of 2–3 sessions being the most common. The total intervention period spanned from 1 to 12 weeks, with most studies (11 studies, 47.8%) implementing a 4−week or 12−week protocol. No serious adverse events were reported in any of the included studies. The Additional details are provided in Table 1.

**Table 1:** Characteristics of included studies.

Study (year, country)	Participants (Age, diagnosis)	Sample size (total, EG, CG)	Intervention group		Control group	Intervention dose			Outcome
			Level of immersion	Virtual reality types		Duration (minute/times)	Frequency (times/week)	Period (week)	
Zhao et al., 2024 (China)	6–12	80 EG:40 CG:40	Non−immersive	Physical activity−based	Psychoeducation	30	3	4	SNAP−IV
Cho et al., 2002 (Korea)	14–18	17 EG:8 CG:9	immersive	Cognitive based	Neurofeedback training	20	4	2	CPT
Cho et al., 2004 (Korea)	14–18	19 EG:10 CG:9	immersive	Cognitive based	No intervention	20	4	2	CPT
Weerdmeester et al., 2016 (Netherlands)	9.77 ± 1.74	73 EG:37 CG:36	Non−immersive	Physical activity−based	Conventional therapy	15	2	3	AVL
Kim et al., 2020 (Korea)	6–10	40 EG:20 CG:20	Non−immersive	Cognitive based	No intervention	30	2.5	6	ATA
Bioulac et al., 2018 (France)	8.9 ± 1.2	35 EG:19 CG:16	immersive	Cognitive based	Methylphenidate	30	2	6	ADHD−RS
Chang et al., 2022 (Taiwan)	8.31 ± 1.20	32 EG:16 CG:16	Non−immersive	Physical activity−based	Conventional therapy	60	3	12	The Stroop Test
Wong et al., 2024 (Hong Kong, China)	6–12	60 EG:30 CG:30	immersive	Cognitive based	Wait−list Control	20	4	3	BRIEF−P
Rodrigo−Yanguas et al., 2023 (Spain)	12–22	70 EG:35 CG:35	immersive	Cognitive based	Active control	60	1	12	SNAP−IV BRIEF−2
Lee et al., 2001 (Korea)	14–18	20 EG:10 CG:10	immersive	Cognitive based	No intervention	10	4	2.5	CPT
Bikic et al., 2017 (Denmark)	15.6 ± 0.99	17 EG:9 CG:8	Non−immersive	Cognitive based	Active control	30	5	7	ADHD−RS
Bikic et al., 2018 (Denmark)	9.96 ± 1.76	70 EG:35 CG:35	Non−immersive	Cognitive based	TUA	30	6	8	ADHD−RS BRIEF−P
Dovis et al., 2015 (Netherlands)	10.6 ± 1.4	61 EG:31 CG:30	immersive	Cognitive based	Placebo	35 ~ 50	5	5	DBDRS−P BRIEF−P
Ji et al., 2023 (China)	8.93 ± 1.54	30 EG:16 CG:14	Non−immersive	Physical activity−based	Active control	50	3	4	FAIR Go/No−go Task
Kim et al., 2024 (Korea)	8–13	23 EG:11 CG:12	immersive	Mixed (Cognitive + Physical)	Psychoeducation	15	2	4	ATA
Hudak et al., 2017 (Germany)	23.40 ± 2.83	20 EG:10 CG:10	immersive	Cognitive based	Active control	60	4	2	Back Go/No−go Task
Molazamani et al., 2023 (Iran)	8.90 ± 1.65	30 EG:15 CG:15	Non−immersive	Cognitive based	Wait−list Control	60	3	4	CPT
Cunha et al., 2023 (Portugal)	20.96 ± 0.99	25 EG:12 CG:13	immersive	Cognitive based	Wait−list Control	30	2	5	SLN
Hahn−Markowitz et al., 2017 (Israel)	8.50 ± 0.85	107 EG:54 CG:53	Non−immersive	Cognitive based	Wait−list Control	60	1	12	BRIEF−P
Benzing et al., 2018 (Switzerland)	10.48 ± 1.38	46 EG:22 CG:24	Non−immersive	Physical activity−based	Watching a video	15	1	1	FTRT Color span task
Benzing et al., 2019 (Switzerland)	10.43 ± 1.37	51 EG:28 CG:23	Non−immersive	Physical activity−based	Conventional therapy	30	3	8	ADHD−RS
Skalski et al., 2021 (Poland)	9–15	60 EG:30 CG:30	immersive	Cognitive based	Traditional neural traditional feedback training	35	1	10	The Stroop Task
Martin−Moratinos, 2024 (Spain)	12.68 ± 2.75	69 EG:33 CG:36	immersive	Cognitive based	Psychoeducation	20	2	12	SNAP−IV

SNAP−IV, Swanson, Nolan, and pelham questionnaire; BRIEF, Behavior rating inventory of executive function; FAIR, Frankfurter aufmerksamkeits−inventar; ATA, Advanced test of attention; SLN, Sequence of letters and numbers; P, Parents; FTRT, Flanker task reaction times; ALA, ADHD Vragen Lijst; CPT, The continuous performance task; ADHD−RS, Attention−deficit/hyperactivity disorder rating scale.

### 3.3 Quality evaluation of included studies

All 23 included studies met the PEDro criteria for specified eligibility, random allocation, baseline comparability, intention−to−treat analysis, between−group statistical comparisons, and point estimates and measures of variability, as shown in Table 1. Among these, nine studies (Dovis et al., 2015; Bikic et al., 2017; Benzing et al., 2018; Bikic et al., 2018; Benzing and Schmidt, 2019; Cunha et al., 2023; Rodrigo−Yanguas et al., 2023; Martin−Moratinos et al., 2024; Wong et al., 2024) adequately implemented allocation concealment, and none reported a participant dropout rate ≤ 15%. According to the PEDro scale, the mean methodological quality score was 6.11, and no study was classified as low quality. Overall, the methodological quality of the included trials was considered good. Detailed results are presented in Supplementary Appendix S2.

According to the ROB−2 assessment, eight studies (Dovis et al., 2015; Cho et al., 2002; Cho et al., 2004; Bikic et al., 2017; Hudak et al., 2017; Benzing et al., 2018; Bikic et al., 2018; Bioulac et al., 2018) clearly described the randomization process and were therefore classified as having a low risk of bias in this domain. Three studies (Dovis et al., 2015; Bioulac et al., 2018; Kim et al., 2020) provided sufficient details regarding intervention protocols and were judged to be at low risk of bias for deviations from intended interventions. All 20 studies showed no evidence of bias due to missing outcome data. Seven studies (Dovis et al., 2015; Benzing et al., 2018; Bioulac et al., 2018; Benzing and Schmidt, 2019; Kim et al., 2020; Cunha et al., 2023; Zhao et al., 2024) were assessed as having a low risk of bias in outcome measurement, and none of the included studies demonstrated selective reporting of outcomes. Only one study (Dovis et al., 2015) was rated as having a low risk of bias across all five domains, resulting in an overall low risk of bias.

Based on the overall ROB−2 judgments, one study (Dovis et al., 2015) was rated as Grade A (low risk of bias across all five domains). Twelve studies (Cho et al., 2002; Cho et al., 2004; Weerdmeester et al., 2016; Bikic et al., 2017; Hudak et al., 2017; Benzing et al., 2018; Bioulac et al., 2018; Kim et al., 2020; Chang et al., 2022; Rodrigo−Yanguas et al., 2023; Wong et al., 2024; Zhao et al., 2024) were rated as Grade B (some concerns in at least one domain). The remaining ten studies (Lee et al., 2001; Hahn−Markowitz et al., 2017; Bikic et al., 2018; Benzing and Schmidt, 2019; Skalski et al., 2021; Cunha et al., 2023; Ji et al., 2023; Molazamani et al., 2023; Kim et al., 2024; Martin−Moratinos et al., 2024) were rated as Grade C (high risk of bias in at least one domain). Study quality was independently assessed by two reviewers (HY and LC). Detailed results are presented in Supplementary Appendix S3.

### 3.4 Meta−analysis

#### 3.4.1 Core symptoms

**Figure 2:**
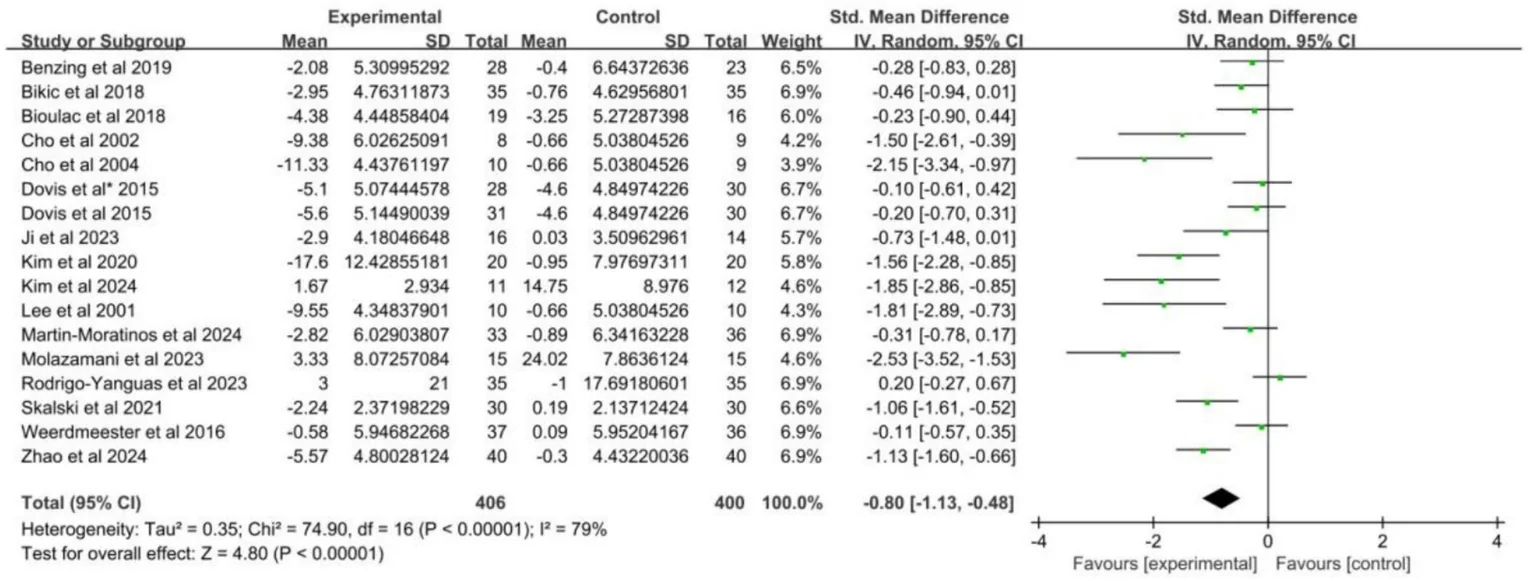
Standard mean differences in inattention between experimental group and control group.

This research evaluated 40 studies (*n* = 2027) investigating the impact of VR on inattention, hyperactivity/impulsivity, and total symptoms in patients with ADHD. The meta−analysis demonstrated significant improvements across all three outcomes:

*Inattention*: Seventeen studies evaluated the effects of VR on inattention in individuals with ADHD. The analysis revealed high heterogeneity (*I^2^* = 79%, *p* < 0.05), necessitating the application of a random−effects model. The pooled effect size was statistically significant [SMD = −0.80, 95% CI: (−1.13, −0.48), *p* < 0.05], indicating a large reduction in inattention levels. The forest plot is presented in Figure 2.

**Figure 3:**
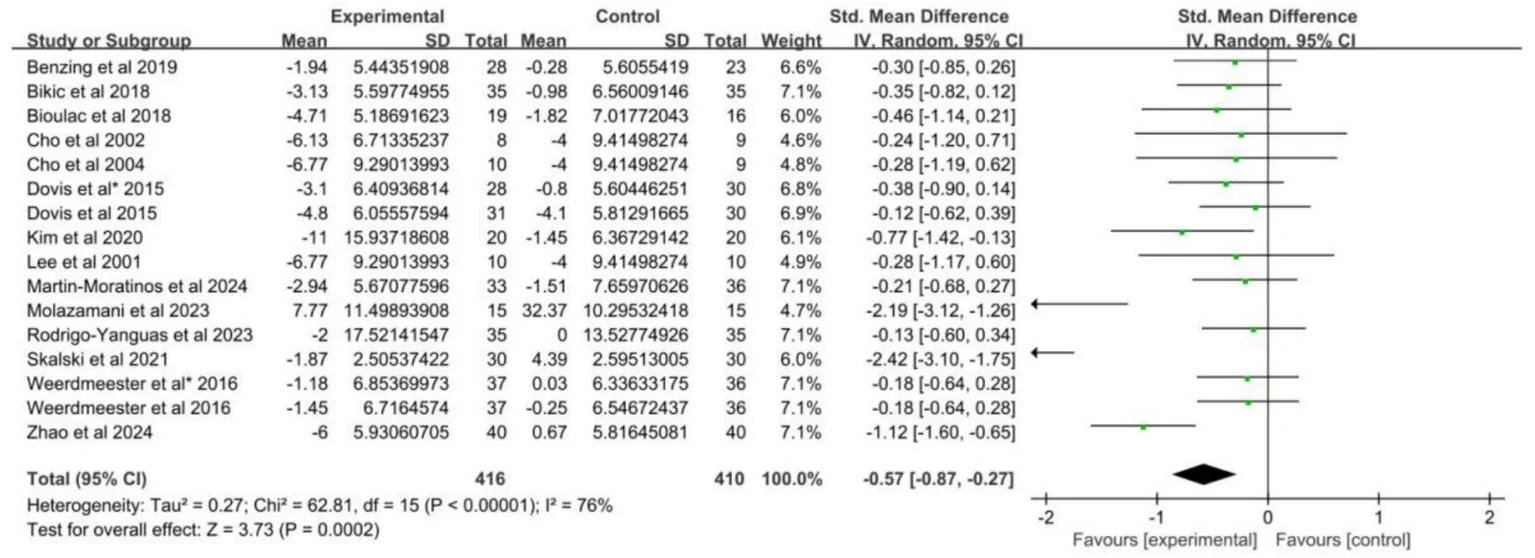
Standard mean differences in hyperactivity/impulsivity between experimental group and control group.

*Hyperactivity/impulsivity*: Sixteen studies evaluated the effects of VR on hyperactivity/impulsivity in individuals with ADHD. The analysis revealed high heterogeneity (*I^2^* = 76%, *p* < 0.05), necessitating the application of a random−effects model. The pooled effect size was statistically significant [SMD = −0.57, 95% CI: (−0.87, −0.27), *p* < 0.05], indicating a moderate reduction in hyperactivity/impulsivity levels. The forest plot is presented in Figure 3.

**Figure 4:**
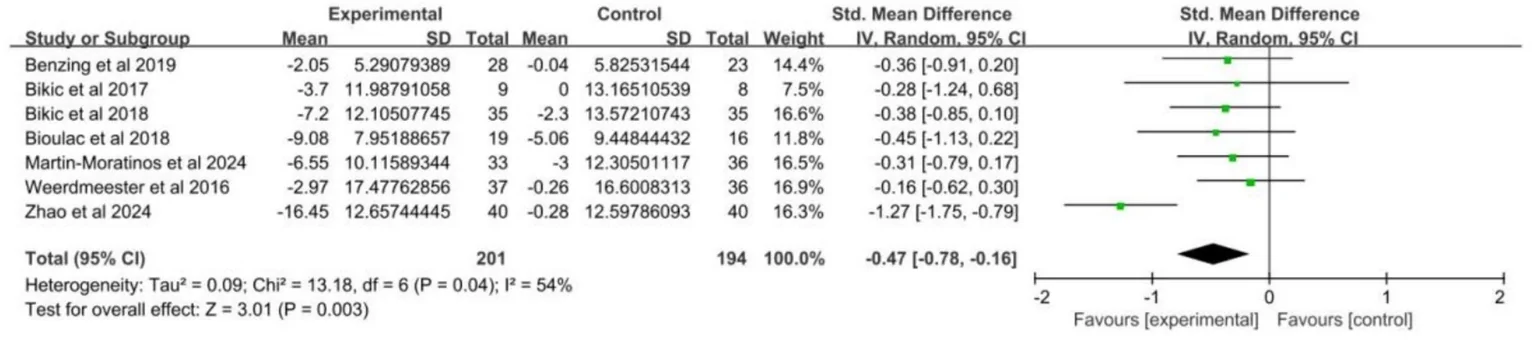
Standard mean differences in total symptoms between experimental group and control group.

*Total symptoms*: Seven studies evaluated the effects of VR on total symptoms in individuals with ADHD. The analysis revealed moderate heterogeneity (*I^2^* = 54%, *p* < 0.05), necessitating the application of a random−effects model. The pooled effect size was statistically significant [SMD = −0.47, 95% CI: (−0.78, −0.16), *p* < 0.05], indicating a small reduction in total symptoms levels. The forest plot is presented in Figure 4.

#### 3.4.2 Cognitive function

**Figure 5:**
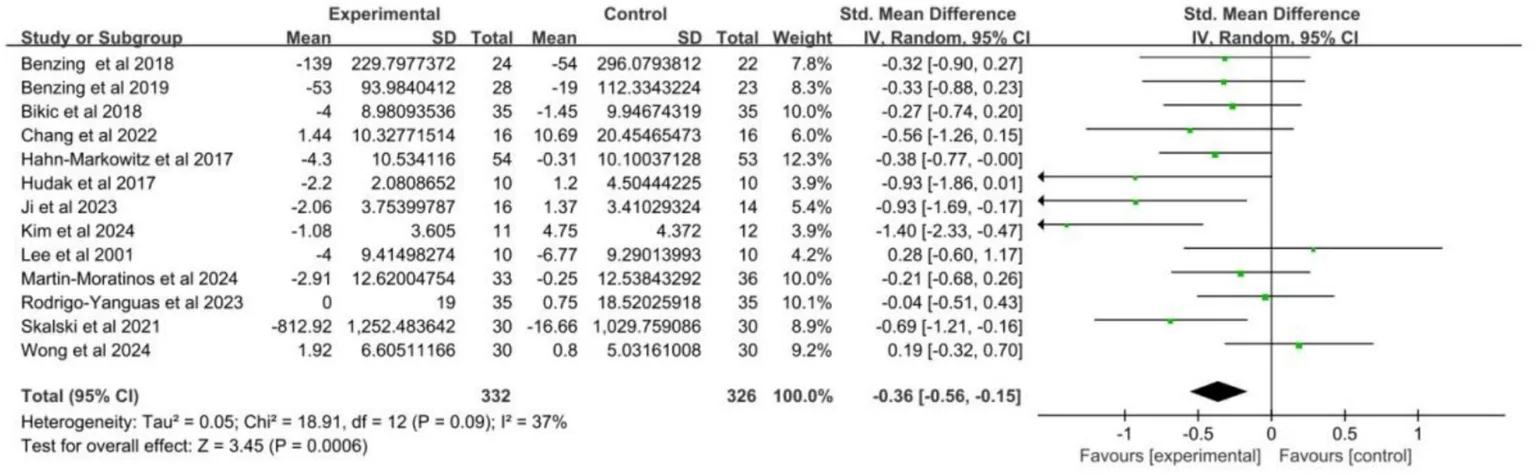
Standard mean differences in inhibition between experimental group and control group.

This research evaluated 29 studies (*n* = 1,650) investigating the impact of VR on inhibition, working memory, and cognitive flexibility in patients with ADHD. The meta−analysis demonstrated significant improvements across all three outcomes:

*Inhibition*: Thirteen studies evaluated the effects of VR on inhibition in individuals with ADHD. The analysis revealed low heterogeneity (*I^2^* = 37%, *p* < 0.05), necessitating the application of a random−effects model. The pooled effect size was statistically significant [SMD = −0.36, 95% CI: (−0.56, −0.15), *p* < 0.05], indicating a small reduction in inhibition levels. The forest plot is presented in Figure 5.

**Figure 6:**
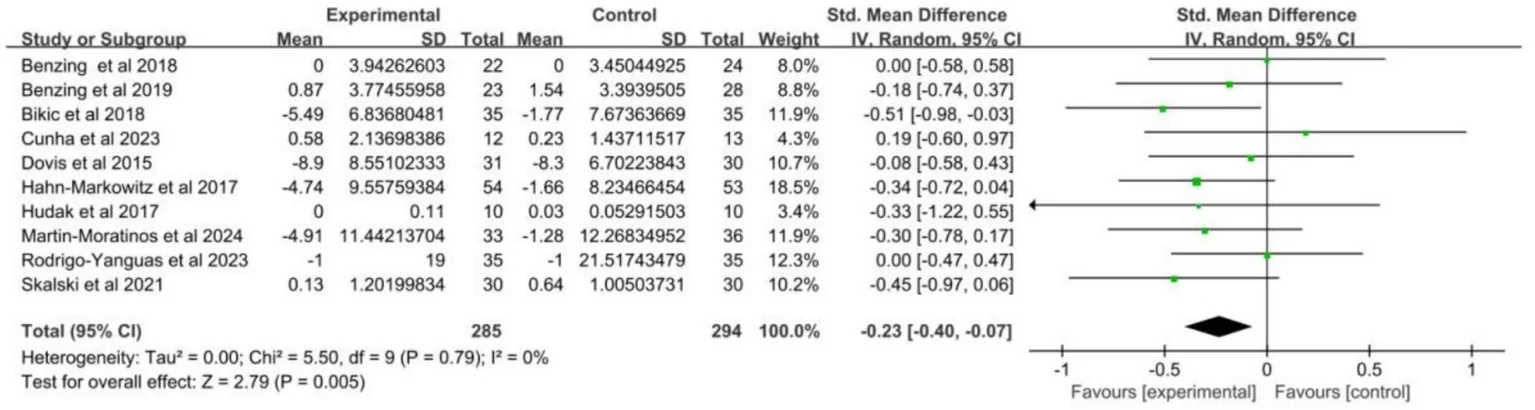
Standard mean differences in working memory between experimental group and control group.

*Working memory*: Ten studies evaluated the effects of VR on working memory in individuals with ADHD. The analysis revealed low heterogeneity (*I^2^* = 0%, p < 0.05), necessitating the application of a random−effects model. The pooled effect size was statistically significant [SMD = −0.23, 95% CI: (−0.40, −0.07), *p* < 0.05], indicating a small reduction in working memory levels. The forest plot is presented in Figure 6.

**Figure 7:**
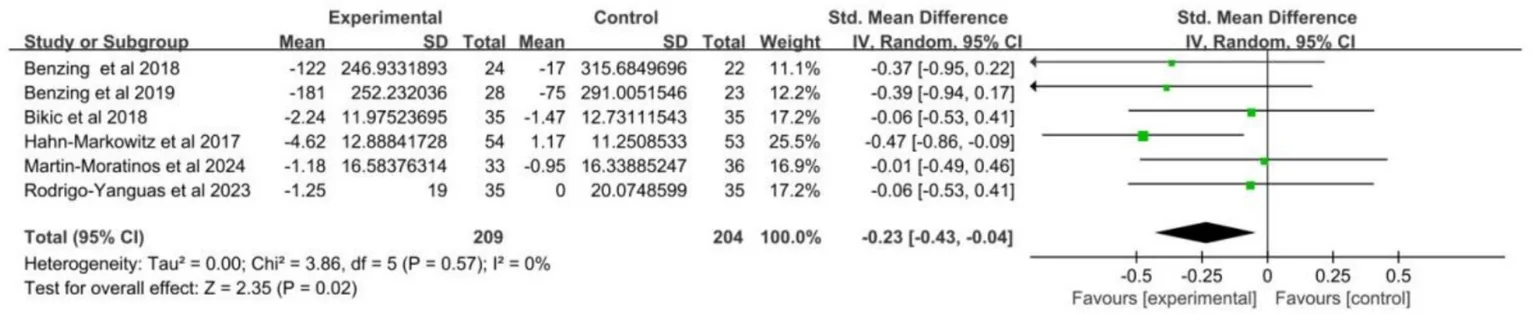
Standard mean differences in cognitive flexibility between experimental group and control group.

*Cognitive flexibility*: Six studies evaluated the effects of VR on cognitive flexibility in individuals with ADHD. The analysis revealed low heterogeneity (*I^2^* = 0%, *p* < 0.05), necessitating the application of a random−effects model. The pooled effect size was statistically significant [SMD = −0.23, 95% CI: (−0.43, −0.04), *p* < 0.05], indicating a small reduction in cognitive flexibility levels. The forest plot is presented in Figure 7.

### 3.5 Subgroup analyses

#### 3.5.1 Subgroup analyses of Core symptoms

Subgroup analyses (Table 2) were performed to investigate the influence of six moderating variables on the efficacy of VR interventions: age, level of immersion, types of Virtual Reality, period/week, frequency, and duration. The findings are summarized as follows:

**Table 2:** Moderator effect results from hierarchical subgroup analysis of core symptoms.

Moderator variables	Subgroup category	Inattention						Hyperactivity/impulsivity						Total symptoms					
		SMD	95% CI	*Z*	*p*	K	N	SMD	95%CI	Z	*p*	K	N	SMD	95%CI	Z	*p*	K	N
Age	<12	−0.56	[−0.86, −0.26]	3.66	0.00	11	590	−0.53	[−0.83, −0.23]	3.49	0.00	10	571	−0.53	[−0.94, −0.12]	2.25	0.01	5	309
	12~18	−0.98	[−1.67, −0.28]	2.77	0.00	6	255	−0.93	[−1.83, −0.02]	2.00	0.05	6	216	−0.30	[−0.73, 0.12]	1.40	0.16	2	86
	≥18	—	—	—	—	—	—	—	—	—	—	—	—	—	—	—	—	—	—
Level of immersion	Immersive	−1.14	[−1.77, −0.51]	3.55	0.00	9	335	−0.52	[−1.04, 0.01]	1.92	0.05	8	351	−0.51	[−0.93, −0.10]	2.41	0.02	5	308
	Non−immersive	−0.78	[−1.24, −0.32]	3.31	0.00	8	432	−0.60	[−0.96, −0.24]	3.29	0.00	8	475	−0.36	[−0.78, 0.07]	1.65	0.10	2	87
Types of virtual reality	Cognitive	−0.85	[−1.27, −0.42]	3.89	0.00	12	549	0.62	[−1.02, −0.23]	3.11	0.00	12	549	−0.36	[−0.64, −0.07]	2.45	0.01	4	191
	physical activity	−0.28	[−0.60, 0.04]	1.73	0.08	3	154	−0.18	[−0.51, 0.15]	1.09	0.28	3	146	−0.24	[−0.59, 0.12]	1.32	0.19	2	124
	Cognitive + physical activity	−1.35	[−2.00, −0.70]	4.80	0.00	2	103	−1.12	[−1.60, −0.65]	4.66	0.00	1	80	−1.27	[−1.75, −0.79]	5.15	0.00	1	80
Min/times (min)	Short (<20 min)	−1.19	[−2.52, 0.13]	1.76	0.08	3	116	−0.19	[−0.50, 0.11]	1.24	0.22	3	166	−0.16	[−0.62, 0.30]	0.67	0.50	1	73
	Moderate (20–30 min)	−0.83	[−1.24, −0.41]	3.89	0.00	8	381	−0.50	[−0.76, −0.24]	3.82	0.00	8	381	−0.54	[−0.88, −0.19]	3.01	0.00	6	322
	Long (>30 min)	−0.69	[−1.25, −0.14]	2.44	0.01	6	312	−1.00	[−1.90, −0.10]	2.19	0.03	5	279	—	—	—	—	—	—
Times/weeks	Low frequency(1~2/week)	−0.63	[−1.13, −0.12]	2.44	0.01	7	370	−0.59	[−1.11, −0.07]	2.24	0.03	7	420	−0.27	[−0.57, 0.02]	1.81	0.07	3	177
	high frequency(≥3/week)	−0.95	[−1.40, −0.50]	4.14	0.00	10	436	−0.55	[−0.92, −0.19]	3.00	0.00	9	406	−0.61	[−1.12, −0.10]	2.35	0.02	4	218
Intervention cycle (weeks)	Short−term (≤4 weeks)	1.39	[−2.00, −0.78]	4.46	0.00	8	292	−0.61	[−1.11, −0.12]	2.44	0.01	7	312	−0.71	[−1.80, 0.38]	1.28	0.20	2	153
	Mid−term (5−8 weeks)	−0.43	[−0.79, −0.07]	2.33	0.02	6	315	−0.36	[−0.59, −0.14]	3.18	0.00	6	315	−0.38	[−0.68, −0.08]	2.45	0.01	4	173
	Long−term (≥9 weeks)	−1.07	[−2.43, 0.29]	1.54	0.12	3	160	−0.90	[−2.17, 0.38]	1.38	0.17	3	199	−0.31	[−0.79, 0.17]	1.28	0.20	1	69

K, number of documents; N, sample size.

*Age*: For children under 12 years of age, significant improvements were observed in inattention (SMD = −0.56), hyperactivity/impulsivity (SMD = −0.53), and total symptoms (SMD = −0.53), all with *p* < 0.05. In adolescents aged 12 to 18 years, significant effects were found for inattention (SMD = −0.98) and hyperactivity/impulsivity (SMD = −0.93) (*p* < 0.05). However, the improvement in total symptoms was not statistically significant (*p* = 0.16). Insufficient data was available for participants aged 18 years and older.

*Level of immersion*: Immersive VR demonstrated significant improvements in inattention (SMD = −1.14), hyperactivity/impulsivity (SMD = −0.52), and total symptoms (SMD = −0.51), all with *p* < 0.05. Non−immersive VR yielded significant improvements in inattention (SMD = −0.78) and hyperactivity/impulsivity (SMD = −0.60) (p < 0.05). However, the reduction in total symptoms was not statistically significant (*p* = 0.10).

*Types of virtual reality*: Cognitive−based VR produced significant improvements in inattention (SMD = −0.85), hyperactivity/impulsivity (SMD = −0.62), and total symptoms (SMD = −0.36), all with *p* < 0.05. Mixed cognitive−physical VR demonstrated significant improvements in inattention (SMD = −1.35), hyperactivity/impulsivity (SMD = −1.12), and total symptoms (SMD = −1.27), all with *p* < 0.05, and yielded the largest effect sizes among all intervention types. But physical activity−based VR did not show statistically significant improvements in any symptom domain (*p* > 0.05).

*VR intervention duration*: Moderate duration (20–30 min): Significant improvements were found in inattention (SMD = −0.83), hyperactivity/impulsivity (SMD = −0.50), and total symptoms (SMD = −0.54), all with *p* < 0.05. Long duration (> 30 min): Significant improvements were observed in inattention (SMD = −0.69) and hyperactivity/impulsivity (SMD = −1.00) (*p* < 0.05). Insufficient data was available for total symptoms. Short duration (< 20 min): No statistically significant improvements were observed in any symptom domain (*p* > 0.05).

*VR intervention frequency*: Low frequency (1–2 sessions per week): Significant improvements were observed in inattention (SMD = −0.63) and hyperactivity/impulsivity (SMD = −0.59) (p < 0.05). However, the reduction in total symptoms was not statistically significant (*p* = 0.07). High frequency (≥ 3 sessions per week): Significant improvements were found in inattention (SMD = −0.95), hyperactivity/impulsivity (SMD = −0.55), and total symptoms (SMD = −0.61), all with *p* < 0.05.

VR intervention cycle: Short−term (≤ 4 weeks): Significant improvements were observed in inattention (SMD = −1.39) and hyperactivity/impulsivity (SMD = −0.61) (*p* < 0.05). However, the reduction in total symptoms was not statistically significant (*p* = 0.20). Mid−term (5–8 weeks): Significant improvements were found in inattention (SMD = −0.43), hyperactivity/impulsivity (SMD = −0.36), and total symptoms (SMD = −0.38), all with *p* < 0.05. Long−term (≥ 9 weeks): No statistically significant improvements were observed in any symptom domain (*p* > 0.05).

#### 3.5.2 Subgroup analyses of cognitive function

Subgroup analyses (Table 3) were performed to investigate the influence of six moderating variables on the efficacy of VR interventions: age, level of immersion, types of Virtual Reality, period/week, frequency, and duration. The findings are summarized as follows:

**Table 3:** Moderator effect results from hierarchical subgroup analysis of cognitive function.

Moderator variables	Subgroup category	Inhibition						Working memory						Cognitive flexibility					
		SMD	95%CI	Z	*p*	K	N	SMD	95%CI	Z	*p*	K	N	SMD	95% CI	Z	*p*	K	N
Age	<12	−0.40	[−0.66, −0.13]	2.93	0.00	8	419	−0.26	[−0.47, −0.04]	2.32	0.02	5	335	−0.33	[−0.57, −0.09]	2.72	0.00	4	274
	12~18	−0.22	[−0.57, 0.13]	1.24	0.21	4	219	−0.24	[−0.52, 0.04]	1.68	0.09	3	199	−0.04	[−0.37, 0.29]	0.23	0.82	2	139
	≥18	−0.93	[−1.86, 0.01]	1.95	0.05	1	20	−0.04	[−0.63, 0.55]	0.14	0.89	2	45		−	−	−	−	−
Level of immersion	Immersive	−0.20	[−0.47, 0.07]	1.47	0.14	7	368	−0.18	[−0.40, 0.05]	1.54	0.12	6	305	−0.33	[−0.57, −0.09]	2.72	0.00	4	274
	Non−immersive	−0.40	[−0.62, −0.18]	3.63	0.00	6	336	−0.30	[−0.53, −0.06]	4.43	0.02	4	274	−0.04	[−0.37, 0.29]	0.23	0.82	2	139
Types of virtual reality	Cognitive	−0.24	[−0.47, −0.02]	2.10	0.04	8	476	−0.26	[−0.44, −0.08]	2.85	0.00	8	482	−3.00	[−6.07, 0.08]	1.91	0.06	4	316
	physical activity	−0.48	[−0.80, −0.15]	2.90	0.00	4	154	−0.10	[−0.50, 0.30]	0.47	0.64	2	97	−105.54	[−216.97, 5.89]	1.86	0.06	2	97
	Cognitive + physical activity	−1.40	[−2.33, −0.47]	2.94	0.00	1	23	—	—	—	—	—	—	—	—	—	—	—	—
Min/times(min)	Short (<20 min)	−0.45	[−1.29, 0.39]	1.06	0.29	3	89	0.00	[−0.58, 0.58]	0.00	1.00	1	46	−0.37	[−0.95, 0.22]	1.23	0.22	1	46
	Moderate (20−30 min)	−0.02	[−0.41, 0.37]	0.11	0.91	2	129	−0.28	[−0.55, −0.01]	2.05	0.04	4	215	−0.13	[−0.42, 0.16]	0.89	0.37	3	190
	Long (>30 min)	−0.41	[−0.60, −0.22]	4.21	0.00	8	440	−0.23	[−0.46, −0.01]	2.08	0.04	5	318	−0.29	[−0.69, 0.11]	1.43	0.15	2	177
Times/weeks	Low frequency(1~2/week)	−0.40	[−0.68, −0.12]	2.83	0.00	6	375	−0.21	[−0.41, −0.01]	2.03	0.04	6	377	−0.25	[−0.48, −0.01]	2.08	0.04	4	292
	high frequency(≥3/week)	−0.31	[−0.63, 0.01]	1.92	0.06	7	283	−0.28	[−0.55, 0.00]	1.94	0.05	4	202	−0.20	[−0.55, 0.16]	1.07	0.28	2	121
Intervention cycle (weeks)	Short−term (≤4 weeks)	−0.46	[−0.98, 0.05]	1.76	0.08	6	199	−0.10	[−0.58, 0.38]	0.40	0.69	2	66	−0.37	[−0.95, 0.22]	1.23	0.22	1	46
	Mid−term (5-8 weeks)	−0.29	[−0.65, 0.07]	1.59	0.11	2	121	−0.21	[−0.49, 0.06]	1.53	0.13	4	207	−0.20	[−0.55, 0.16]	1.07	0.28	2	121
	Long−term (≥9 weeks)	−0.34	[−0.56, −0.13]	3.11	0.00	5	338	−0.28	[−0.50, −0.05]	2.39	0.02	4	306	−0.04	[−0.37, 0.29]	0.23	0.82	2	139

K, number of documents; N, sample size.

*Age*: For children under 12 years of age, significant improvements were observed in inhibition (SMD = −0.40), working memory (SMD = −0.26), and cognitive flexibility (SMD = −0.33), all with *p* < 0.05. For adolescents aged 12 to 18 years, no statistically significant improvements were found in any of the executive function domains (*p* > 0.05). For participants aged 18 years and older, the improvement in inhibition approached statistical significance (SMD = −0.93, *p* = 0.05). No significant improvement was observed in working memory (*p* = 0.89), and insufficient data were available for cognitive flexibility.

*Level of immersion*: Immersive VR did not yield statistically significant improvements in inhibition, working memory, or cognitive flexibility (*p* > 0.05), except for a significant improvement in cognitive flexibility under immersive conditions (SMD = −0.33, *p* < 0.05). Non−immersive VR demonstrated significant improvements in inhibition (SMD = −0.40) and working memory (SMD = −0.30) (*p* < 0.05), but no significant improvement was observed in cognitive flexibility (SMD = −0.04, *p* = 0.82).

*Types of virtual reality*: Cognitive−based VR significantly improved inhibition (SMD = −0.24) and working memory (SMD = −0.26) (*p* < 0.05). The improvement in cognitive flexibility approached statistical significance (SMD = −3.00, *p* = 0.06). Physical activity−based VR significantly improved inhibition (SMD = −0.48, *p* < 0.05). However, the improvements in working memory and cognitive flexibility were not statistically significant (p > 0.05). Mixed cognitive−physical VR was investigated in only one study, which showed a significant improvement in inhibition (SMD = −1.40, *p* < 0.05). Insufficient data were available for working memory and cognitive flexibility.

*VR intervention duration*: Short duration (< 20 min): No statistically significant improvements were observed in any executive function domain (*p* > 0.05). Moderate duration (20–30 min): A significant improvement was found in working memory (SMD = −0.28, *p* = 0.04). However, no significant improvements were observed in inhibition or cognitive flexibility (*p* > 0.05). Long duration (> 30 min): Significant improvements were observed in inhibition (SMD = −0.41) and working memory (SMD = −0.23) (*p* < 0.05). No significant improvement was found in cognitive flexibility (*p* = 0.15).

*VR intervention frequency*: Low frequency (1–2 sessions per week) was associated with significant improvements in inhibition (SMD = −0.40), working memory (SMD = −0.21), and cognitive flexibility (SMD = −0.25), all with *p* < 0.05. High frequency (≥ 3 sessions per week) showed no statistically significant improvements (*p* = 0.06 for inhibition, p = 0.05 for working memory, and *p* = 0.28 for cognitive flexibility), with the effects being either non−significant or only approaching significance, thus remaining inconclusive.

VR intervention cycle: Long−term intervention (≥ 9 weeks) resulted in significant improvements in inhibition (SMD = −0.34) and working memory (SMD = −0.28) (*p* < 0.05). However, no significant improvement was observed in cognitive flexibility (*p* = 0.82). Short−term (≤ 4 weeks) and mid−term (5–8 weeks) interventions did not yield statistically significant improvements in any of the executive function domains (*p* > 0.05).

#### 3.5.3 Sensitivity analysis

**Figure 8:**
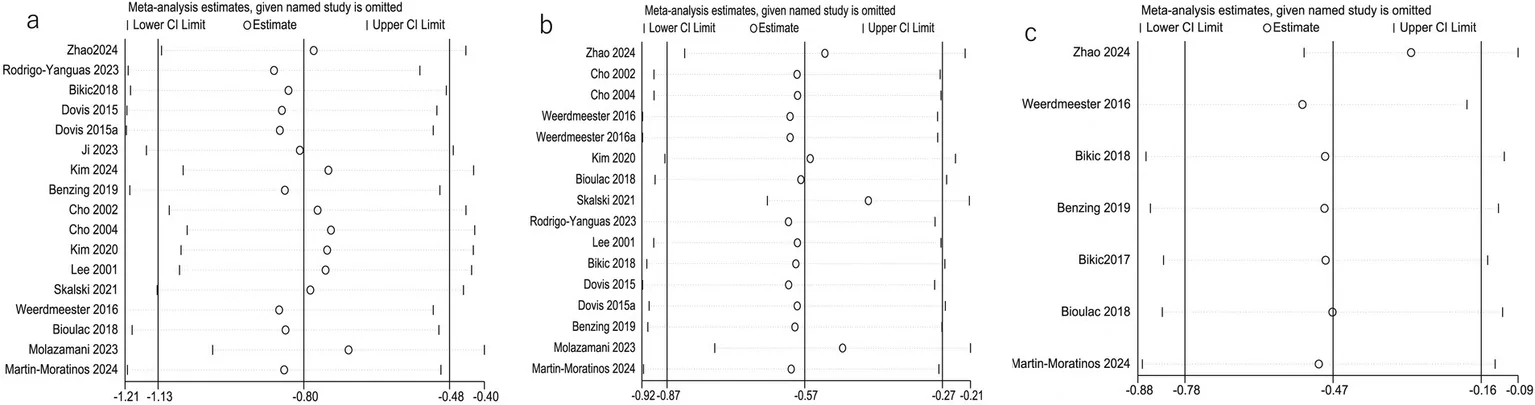
Sensitivity analysis [**(a)** inattention; **(b)** hyperactivity/impulsivity; **(c)** total symptoms].

To investigate the source of heterogeneity, a sensitivity analysis was performed using Stata 18.0. As illustrated in Figure 8, the combined effect was analyzed by sequentially excluding individual studies. After removing individual studies, the results for inattention revealed SMD (−0.74 to −0.87), *I^2^* (74 –80%), and *p* < 0.05; for hyperactivity/impulsivity, SMD (−0.43 to −0.60), *I^2^* (53 –78%), and *p* < 0.05; and for total symptoms, SMD (−0.31 to −0.54), *I^2^* (0 –62%), and *p* < 0.05. The analysis demonstrated no significant changes before and after the removal of individual studies. The low sensitivity of the study data indicates that the results exhibit a considerable level of reliability and stability. These findings suggest that the combined analysis did not result in substantial alterations, thus demonstrating a degree of robustness. As the heterogeneity for all cognitive subdomains was low to moderate, no additional sensitivity analysis was performed. The heterogeneity for each subdomain of cognitive function was low (0−37%), no further sensitivity analysis was performed.

### 3.6 Publication bias

Egger’s test revealed no significant differences in hyperactivity/impulsivity (*Z* = −2.25, *p* > |*t*| = 0.155 > 0.05), total symptoms (*Z* = −1.65, *p* > |t| = 0.838 > 0.05), inhibition (*Z* = −1.59, *p* > |*t*| = 0.134 > 0.05), working memory (*Z* = 0.98, *p* > |*t*| = 0.306 > 0.05), or cognitive flexibility (Z = 0.19, *p* > |*t*| = 0.766 > 0.05). These results indicated that the findings were relatively robust.

Egger’s test revealed significant differences in inattention (*Z* = −4.43, *p* > |*t*| = 0.001 < 0.05), to further assess the impact of potential publication bias, a nonparametric trim−and−fill analysis was conducted. The pooled effect size for inattention decreased from Hedges’s g = −0.841 (95% CI, −1.214 to −0.468) to g = −0.544 (95% CI, −0.979 to −0.110) after adjustment. Although the effect size was attenuated, it remained statistically significant, suggesting that while publication bias may be present, the overall conclusion of a beneficial effect of VR interventions on inattention is relatively robust.

### 3.7 Quality of evidence assessment

**Figure 9:**
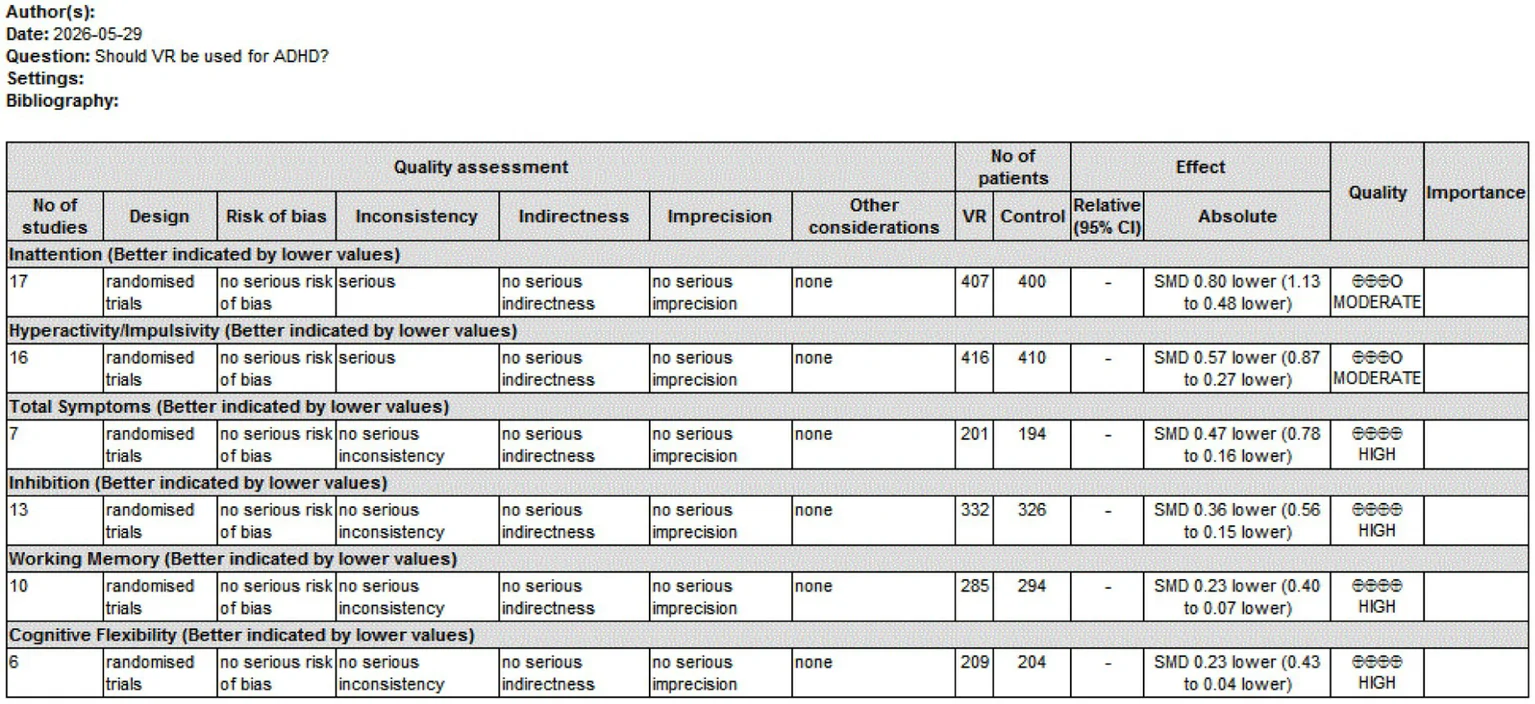
Evidence quality assessment.

The quality of evidence was evaluated utilizing the GRADE profiler methodology. For inattention, the evidence quality was rated as moderate due to serious inconsistency, although no other downgrading factors were identified. For hyperactivity/impulsivity, the evidence quality was also rated as moderate, downgraded for serious inconsistency. For total symptoms, inhibition, working memory, and cognitive flexibility, no downgrading factors were identified in terms of risk of bias, inconsistency, indirectness, or imprecision. Therefore, the evidence quality for these outcomes was rated as high. The assessment results are presented in Figure 9.

## 4 Discussion

This systematic review and meta−analysis included 23 randomized controlled trials involving 1,102 patients with ADHD, which systematically evaluated the effects of virtual reality (VR) interventions on core symptoms (inattention, hyperactivity/impulsivity, and total symptoms) and cognitive functions (inhibition, working memory, and cognitive flexibility). To our knowledge, this is the first meta−analysis to simultaneously examine the effects of VR interventions on both core symptoms and cognitive functions in ADHD. The results showed that, for core symptoms, VR interventions significantly improved inattention (SMD = −0.80, 95% CI: −1.13 to −0.48, moderate−to−large effect), hyperactivity/impulsivity (SMD = −0.57, 95% CI: −0.87 to −0.27, moderate effect), and total symptoms (SMD = −0.47, 95% CI: −0.78 to −0.16, small−to−moderate effect), with moderate to high quality of evidence. Regarding cognitive functions, VR interventions significantly enhanced inhibition (SMD = −0.36, 95% CI: −0.56 to −0.15, small effect), working memory (SMD = −0.23, 95% CI: −0.40 to −0.07, small effect), and cognitive flexibility (SMD = −0.23, 95% CI: −0.43 to −0.04, small effect), with high quality of evidence. These findings indicate that VR interventions not only alleviate core ADHD symptoms but also improve executive functions to a certain extent, with consistent effect directions favoring the experimental group. As an emerging non−pharmacological intervention, VR holds promising prospects for application in ADHD.

The findings of this study are consistent with those of previous meta−analyses. (Hamada et al., 2025) reported a pooled effect size of SMD = −0.40 for computer game−based interventions on inattention and hyperactivity/impulsivity. The effect sizes in the present study (SMD = −0.80 and −0.57) were slightly higher, which may be attributed to the greater immersion, enhanced ecological validity, and higher patient engagement associated with VR technology. (Sun et al., 2025), in their systematic review of VR−based exercise interventions, also reported positive effects of VR on executive function subdomains, including inhibitory control, attention, working memory, switching, and planning. The present study further confirms these findings and, for the first time, simultaneously covers both core symptoms and cognitive functions within the same meta−analysis, thereby providing a more comprehensive evidence base for the holistic evaluation of VR interventions.

Compared to traditional cognitive training (e.g., Cogmed), the effect sizes in the present study were comparable or slightly superior. (Dovis et al., 2015) reported an improvement in inhibition function (SMD = −0.36) following multi−executive function training, which is exactly consistent with the effect size for inhibitory control found in the present study (SMD = −0.36). However, traditional cognitive training typically yields larger effect sizes for working memory (SMD = −0.50), whereas the VR intervention in the present study showed only a small effect on working memory (SMD = −0.23), suggesting that VR may be less effective than more targeted cognitive training in improving working memory.

The potential mechanisms by which VR interventions may improve core symptoms and cognitive functions in children with ADHD include the following. First, children with ADHD often have low tolerance for delayed rewards and repetitive tasks. The immediate feedback and engaging nature of VR interventions may enhance treatment adherence (Cao et al., 2026; Riva et al., 2019), as supported by the high compliance rates (over 85%) reported in most included studies. Second, some VR interventions integrate physical activity (e.g., exergaming or combined motor−cognitive training). Subgroup analyses revealed that mixed cognitive−physical VR interventions yielded the strongest improvements in core symptoms (inattention: SMD = −1.35; hyperactivity/impulsivity: SMD = −1.12). This suggests that physical activity may augment the effects of VR through neurobiological mechanisms, such as increasing dopamine and norepinephrine levels and promoting prefrontal cortex activation. Third, VR tasks require continuous engagement of cognitive processes including attention, memory updating, rule switching, and inhibitory control. Repeated practice may enhance the functional efficiency of relevant neural circuits (e.g., prefrontal−parietal network and anterior cingulate cortex) (Parsons and Rizzo, 2008; Freeman et al., 2017). Subgroup analyses indicated that long−term interventions (≥9 weeks) produced the most pronounced improvements in inhibitory control and working memory, consistent with the principle that neuroplasticity requires sufficient cumulative training. Fourth, physical activity involved in VR interventions has been shown to enhance prefrontal activation, increase brain−derived neurotrophic factor (BDNF) secretion, and improve inhibitory control and working memory (Chang et al., 2022). In this study, physical activity−based VR produced a larger effect on inhibitory control (SMD = −0.48) than cognitive−based VR (SMD = −0.24), further supporting the unique role of physical activity in cognitive enhancement. Notably, although the pooled effect sizes for cognitive functions (SMD: −0.23 to −0.36) were smaller than those for core symptoms, the heterogeneity was very low (*I^2^*: 0 to 37%), indicating more stable and consistent effects of VR on cognitive functions across studies.

This study is the first to systematically compare the effects of different intervention parameters on core symptoms and cognitive functions, revealing distinct dose–response characteristics for the two outcomes. This finding has important clinical implications. The results of subgroup analyses showed that Core symptoms respond optimally to mid−term (5–8 weeks), moderate−duration (20–30 min), high−frequency (≥3 sessions/week) interventions using mixed cognitive−physical or cognitive−based VR in children under 12 years, with immersive VR producing more consistent broad symptom relief. Executive functions require long−term (≥9 weeks) interventions with long duration (>30 min) per session. Lower frequency (1–2 sessions/week) appears more beneficial than higher frequency, and non−immersive VR may be more effective than immersive for inhibition and working memory. Cognitive improvements are most evident in children under 12 years. These findings highlight different dose–response profiles for symptomatic versus cognitive outcomes, indicating that intervention protocols should be tailored according to the primary target (core symptom reduction vs. executive function enhancement).

The significant heterogeneity in core symptoms, inattention (*I^2^* = 79%, *p* < 0.05), hyperactivity/impulsivity (*I^2^* = 76%, *p* < 0.05), total symptoms (*I^2^* = 54%, *p* < 0.05), which may be related to differences across included studies varied in intervention duration (2–24 weeks), frequency (1–4 sessions per week), and session length. Both immersive and non−immersive VR devices were used. Outcome measures differed across studies, with various rating scales or questionnaires employed to assess symptoms. Additionally, the age range (8–15 years) and other demographic characteristics of participants varied. Future research should consider conducting subgroup analyses or meta−regression on these potential moderating factors to clarify which intervention characteristics or sample features are associated with better intervention outcomes.

The main strengths of this study are as follows: First, only RCTs were included, which is the most reliable study design for evaluating intervention effects, ensuring the credibility of the study conclusions. Second, multiple databases were searched, and two researchers independently completed literature screening and data extraction, reducing subjective bias. Third, both core symptoms and cognitive functions were examined simultaneously, providing a more comprehensive assessment of the effects of VR interventions. Fourth, the robustness of the results was verified through sensitivity analysis. This study is the first to systematically distinguish the differential response patterns of core symptoms and cognitive functions to VR interventions, revealing differences in optimal intervention parameters between the two outcome domains. This finding provides an important reference for the design of future studies and offers valuable directions for mechanistic research on VR interventions.

### 4.1 Limitations

The limitations of this study also require careful consideration. First, the number of included studies was limited, and the total sample size was modest (1,102 participants), which restricted our ability to conduct more in−depth analyses, such as reliably testing for publication bias (Sterne et al., 2011), and adequately analyzing factors that may influence intervention effects. Second, considerable heterogeneity existed among the included studies, which to some extent affected the precision and generalizability of the results. Third, most studies had methodological issues, particularly insufficient description of the randomization process, which may affect the overall quality of the evidence. Fourth, none of the studies conducted follow−up assessments, so we do not know how long the intervention effects may last. Fifth, only English−language published studies were included, which may omit relevant research published in other languages.

Based on the above analysis, several recommendations for future research are proposed: First, conduct larger scale, more rigorously designed RCTs and report them according to international standards to improve research quality. Second, attempt to analyze factors that may influence intervention effects, such as which intervention protocols are more effective and which children are more likely to benefit. Third, standardized measurement tools for core symptoms and cognitive functions are needed. Fourth, incorporate follow−up assessments into study designs (such as 6 months and 12 months after intervention completion) to examine whether effects can be maintained long−term. Fifth, combining neuroimaging (e.g., FNIRS, fMRI) or electrophysiological measures (e.g., EEG, ERP) with physiological indicators (e.g., heart rate variability, galvanic skin response) can help elucidate the mechanisms of VR interventions on brain function and the autonomic nervous system. Moreover, assessing the feasibility, acceptability, and cost−effectiveness of VR interventions in authentic clinical settings is essential for translating research findings into clinical application.

## 5 Conclusion

This systematic review and meta−analysis demonstrate that VR interventions significantly improve core symptoms (inattention, hyperactivity/impulsivity) and select cognitive functions (inhibitory control, working memory, cognitive flexibility) in children with ADHD. Core symptoms respond more rapidly to VR interventions with larger effect sizes, whereas improvements in cognitive functions require longer−term training, lower frequency, and longer session duration. Mixed cognitive−physical VR, immersive VR, and short−to−medium−term, high−frequency interventions are more effective for improving core symptoms, while non−immersive VR and long−term, low−frequency interventions are more suitable for enhancing executive functions. Although the current evidence shows positive effects, the findings should be interpreted with caution due to limitations including sample size, heterogeneity, methodological quality, and lack of follow−up assessments. Future high−quality, long−term follow−up RCTs with well−controlled intervention parameters are needed to further validate the effects of VR interventions, identify optimal intervention parameters, elucidate underlying mechanisms, and facilitate the translation of VR from research to clinical practice.

## Data Availability

The original contributions presented in the study are included in the article/Supplementary material, further inquiries can be directed to the corresponding author.
