# Supplementary material for: Effectiveness of virtual reality based intervention on core symptoms and cognitive functions in attention−deficit hyperactivity disorder: a systematic review and meta−analysis
Source: Front Psychol. 2026 Aug 7;17:1835922. doi: 10.3389/fpsyg.2026.1835922 (PMC13494754; doi:10.3389/fpsyg.2026.1835922)
Supplement: Supplementary file 1 [file Supplementary_file_1.docx]

**Supplementary Materials:**

**Appendix 1**

**Table 1: Search Strategy**

| **Database** | **Search Strategy** |
| --- | --- |
| Cochrane  and  Pubmed | #1 “virtual reality”[Mesh] OR “VR” [Title/Abstract] OR “Reality, Virtual” [Title/Abstract] OR “virtual reality” [Title/Abstract] OR “game based virtual reality” [Title/Abstract] OR “computer based virtual reality” [Title/Abstract] OR “active video games” [Title/Abstract] OR “virtual game” [Title/Abstract] OR “virtual environment” [Title/Abstract]  #2 “Attention Deficit Disorder with Hyperactivity” [Mesh] OR “Attention Deficit Disorder with Hyperactivity” [Title/Abstract] OR “attention deficit hyperactivity disorder” [Title/Abstract] OR “ADHD” [Title/Abstract] OR “Attention Deficit Disorder” [Title/Abstract] OR “ADDH” [Title/Abstract] OR “Hyperkinetic Syndrome” [Title/Abstract]  #3 #1 AND #2 |
| Embase | 1 “virtual reality” [exp] OR “VR”[ab,ti]OR “Reality, Virtual” [ab,ti] OR “virtual reality” [ab,ti] OR “game based virtual reality” [ab,ti] OR “computer based virtual reality” [ab,ti] OR “active video games” [ab,ti] OR “virtual game” [ab,ti] OR “virtual environment” [ab,ti]  #2 “attention deficit hyperactivity disorder” [exp] OR “Attention Deficit Disorder with Hyperactivity” [ab,ti] OR  “attention deficit hyperactivity disorder” [ab,ti] OR “ADHD”[ab,ti] OR “Attention Deficit Disorder” [ab,ti] OR “ADDH” [ab,ti] OR “Hyperkinetic Syndrome” [ab,ti]  #3 #1 AND #2 |
| Web of  Science | #1 TS=（ “virtual reality” OR “VR” OR “Reality, Virtual” OR “game based virtual reality” OR “computer based virtual reality” OR “active video games” OR “virtual game” OR “virtual environment” ）  #2 TS=（ “attention deficit hyperactivity disorder” OR “Attention Deficit Disorder with Hyperactivity” OR “ADHD” OR “Attention Deficit Disorder” OR “ADDH” OR “Hyperkinetic Syndrome”  #3 #1 AND #2 |
| ProQuest | #1 SU.EXACT.EXPLODE("Virtual Reality") OR TI,AB("virtual reality" OR vr OR "virtual environment*" OR "immersive reality" OR "exergam*" OR "serious gam*" OR "active video game*" OR "digital therapeutic*") OR TI,AB("virtual" NEAR/3 (game* OR intervention* OR training))  #2 SU.EXACT.EXPLODE("Attention Deficit Hyperactivity Disorder") OR TI,AB("attention deficit*" OR "attention-deficit/hyperactivity disorder" OR adhd OR addh OR "hyperkinetic syndrome" OR "hyperkinetic disorder" OR "minimal brain dysfunction") OR TI,AB((attenti* NEAR/3 deficit*) OR (hyperkin* NEAR/2 disorder*))  #3 #1 AND #2 |
| EBSCOhost | (TI ("virtual reality" OR "VR" OR "virtual environment*" OR "active video game*" OR "exergam*" OR "serious game*") OR AB ("virtual reality" OR "VR" OR "virtual environment*" OR "active video game*" OR "exergam*" OR "serious game*")) AND (TI ("attention deficit hyperactivity disorder" OR "ADHD" OR "attention deficit disorder" OR "ADDH" OR "hyperkinetic syndrome") OR AB ("attention deficit hyperactivity disorder" OR "ADHD" OR "attention deficit disorder" OR "ADDH" OR "hyperkinetic syndrome")) |

**Appendix 2**

**Table 2 Methodological quality of the included studies**

| **Included study** | **A1** | **A2** | **A3** | **A4** | **A5** | **A6** | **A7** | **A8** | **A9** | **A10** | **A11** | **Toal score** |
| --- | --- | --- | --- | --- | --- | --- | --- | --- | --- | --- | --- | --- |
| Zhao et al., 2024 | Y | Y | N | Y | N | N | N | N | Y | Y | Y | 5/10 |
| Cho et al., 2002 | Y | Y | N | Y | N | N | N | Y | N | Y | Y | 6/10 |
| Cho et al., 2004 | Y | Y | N | Y | N | N | N | Y | N | Y | Y | 6/10 |
| Weerdmeester et al., 2016 | Y | Y | N | Y | N | N | Y | Y | Y | Y | Y | 7/10 |
| Kim et al., 2020 | Y | Y | N | Y | N | N | N | Y | N | Y | Y | 5/10 |
| Bioulac et al., 2018 | Y | Y | N | Y | N | N | N | Y | N | Y | Y | 5/10 |
| Chang et al., 2022 | Y | Y | N | Y | N | N | Y | Y | N | Y | Y | 6/10 |
| Wong et al., 2024 | Y | Y | Y | Y | N | N | Y | Y | Y | Y | Y | 8/10 |
| Rodrigo-Yanguas et al.,2023 | Y | Y | Y | Y | N | N | N | Y | Y | Y | Y | 7/10 |
| Lee et al., 2001 | Y | Y | N | Y | N | Y | N | Y | N | Y | Y | 6/10 |
| Bikic et al.,2017 | Y | Y | Y | Y | Y | N | Y | Y | N | Y | Y | 8/10 |
| Bikic et al., 2018 | Y | Y | Y | Y | N | N | Y | Y | Y | Y | Y | 8/10 |
| Dovis et al., 2015 | Y | Y | Y | Y | Y | Y | Y | Y | Y | Y | Y | 5/10 |
| Ji et al., 2023 | Y | Y | N | Y | N | N | N | Y | N | Y | Y | 5/10 |
| Kim et al., 2024 | Y | Y | N | Y | N | N | N | Y | N | Y | Y | 5/10 |
| Hudak et al., 2017 | Y | Y | N | Y | Y | N | N | Y | N | Y | Y | 6/10 |
| Molazamani et al., 2023 | Y | Y | N | Y | N | N | N | Y | N | Y | Y | 5/10 |
| Cunha et al., 2023 | Y | Y | Y | Y | N | N | Y | Y | N | Y | Y | 5/10 |
| Hahn-Markowitz et al., 2017 | Y | Y | N | Y | N | N | N | Y | N | Y | Y | 5/10 |
| Benzing et al., 2018 | Y | Y | Y | Y | Y | N | N | Y | N | Y | Y | 7/10 |
| Benzing et al., 2019 | Y | Y | Y | Y | Y | N | N | Y | Y | Y | Y | 8/10 |
| Skalski et Yal., 2021 | Y | Y | N | Y | N | N | N | Y | N | Y | Y | 5/10 |
| Martin-Moratinos et al., 2024 | Y | Y | Y | Y | N | N | N | Y | Y | Y | Y | 7/10 |

Abbreviations: N, no; Y, yes; A1, participant selection criteria speciﬁed; A2, random assignment of participants to groups; A3, hidden assignment; A4, groups were similar at baseline; A5, all participants were blind; A6, all therapists were blind; A7, all assessors were blind; A8, measurement of at least one of the main outcomes was obtained from more than 85% of the participants; A9, intention-to-treat analysis was conducted; A10, results of statistical comparisons between groups for at least one main outcome were reported; A11, the study reported point and variability measures for at least one main outcome.

**Appendix 3**

**Table 3 . Risk of bias assessment**

| **Study** | **Randomization process** | **Deviation from expected intervention** | **Missing outcome data** | **Outcome measurement** | **Selection of report results** | **Overall** |
| --- | --- | --- | --- | --- | --- | --- |
| Zhao et al., 2024 | Some concerns | Low risk | Low risk | Low risk | Low risk | Some concerns |
| Cho et al., 2002 | Low risk | Low risk | Some concerns | Low risk | Low risk | Some concerns |
| Cho et al., 2004 | Low risk | Low risk | Low risk | Some concerns | Low risk | Some concerns |
| Weerdmeester et al., 2016 | Some concerns | Low risk | Low risk | Some concerns | Low risk | Some concerns |
| Kim et al., 2020 | Low risk | Low risk | Low risk | Low risk | Low risk | Low risk |
| Bioulac et al., 2018 | Low risk | Low risk | Low risk | Low risk | Low risk | Low risk |
| Chang et al., 2022 | Low risk | Some concerns | Low risk | Low risk | Low risk | Some concerns |
| Rodrigo-Yanguas et al., 2023 | Some concerns | Low risk | Low risk | Some concerns | Low risk | Some concerns |
| Wong et al., 2024 | Some concerns | Some concerns | Low risk | Low risk | Low risk | Some concerns |
| Lee et al., 2001 | High risk | High risk | Low risk | Low risk | Some concerns | Some concerns |
| Bikic et al.,2017 | Low risk | Low risk | Some concerns | Low risk | Low risk | Some concerns |
| Bikic et al., 2018 | Low risk | High risk | Low risk | Some concerns | Low risk | Some concerns |
| Dovis et al., 2015 | Low risk | Low risk | Low risk | Low risk | Low risk | Low risk |
| Ji et al., 2023 | Some concerns | High risk | Some concerns | Some concerns | Some concerns | Some concerns |
| Kim et al., 2024 | Some concerns | High risk | Some concerns | Some concerns | Low risk | Some concerns |
| Hudak et al., 2017 | Low risk | Low risk | Low risk | Some concerns | Low risk | Some concerns |
| Molazamani et al., 2023 | Some concerns | High risk | Low risk | Some concerns | Low risk | Some concerns |
| Cunha et al., 2023 | Low risk | High risk | Low risk | Low risk | Low risk | Some concerns |
| Hahn-Markowitz et al., 2017 | Low risk | High risk | Low risk | Some concerns | Low risk | Some concerns |
| Benzing et al., 2018 | Low risk | Low risk | Some concerns | Low risk | Low risk | Some concerns |
| Benzing et al., 2019 | Low risk | High risk | Low risk | Some concerns | Low risk | Some concerns |
| Skalski et Yal., 2021 | Some concerns | High risk | Low risk | Some concerns | Low risk | Some concerns |
| Martin-Moratinos et al., 2024 | Low risk | High risk | Low risk | High risk | Low risk | Some concerns |
